# Adduct Ions as Diagnostic Probes of Metallosupramolecular Complexes Using Ion Mobility Mass Spectrometry

**DOI:** 10.1021/acs.inorgchem.2c03698

**Published:** 2023-01-30

**Authors:** Niklas Geue, Tom S. Bennett, Lennart A. I. Ramakers, Grigore A. Timco, Eric J. L. McInnes, Neil A. Burton, P. B. Armentrout, Richard E. P. Winpenny, Perdita E. Barran

**Affiliations:** † Michael Barber Centre for Collaborative Mass Spectrometry, Manchester Institute of Biotechnology, Department of Chemistry, The University of Manchester, 131 Princess Street, Manchester M1 7DN, UK; ‡ Department of Chemistry, The University of Manchester, Oxford Road, Manchester M13 9PL, UK; § Department of Chemistry, University of Utah, Salt Lake City, Utah 84112, United States

## Abstract

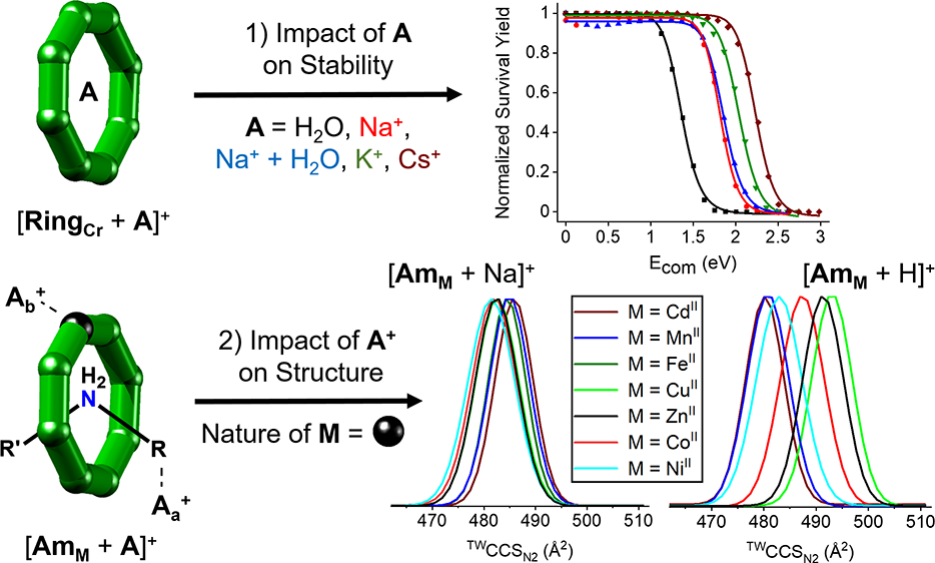

Following electrospray ionization, it is common for analytes to enter the gas phase accompanied by a charge-carrying ion, and in most cases, this addition is required to enable detection in the mass spectrometer. These small charge carriers may not be influential in solution but can markedly tune the analyte properties in the gas phase. Therefore, measuring their relative influence on the target molecule can assist our understanding of the structure and stability of the analyte. As the formed adducts are usually distinguishable by their mass, differences in the behavior of the analyte resulting from these added species (e.g., structure, stability, and conformational dynamics) can be easily extracted. Here, we use ion mobility mass spectrometry, supported by density functional theory, to investigate how charge carriers (H^+^, Na^+^, K^+^, and Cs^+^) as well as water influence the disassembly, stability, and conformational landscape of the homometallic ring [Cr_8_F_8_(O_2_C^*t*^Bu)_16_] and the heterometallic rotaxanes [NH_2_RR′][Cr_7_MF_8_(O_2_C^*t*^Bu)_16_], where M = Mn^II^, Fe^II^, Co^II^, Ni^II^, Cu^II^, Zn^II^, and Cd^II^. The results yield new insights on their disassembly mechanisms and support previously reported trends in cavity size and transition metal properties, demonstrating the potential of adduct ion studies for characterizing metallosupramolecular complexes in general.

## Abstract 2

Using ion mobility mass spectrometry, we show how different charge-carrying ions and small molecules can tune the stability and conformational landscape of metallosupramolecular complexes and aid their structural characterization.

## Introduction

Mass spectrometry (MS) allows the investigation of ions in the gas phase and, in combination with advanced fragmentation techniques, enables the analysis of their disassembly. The most common fragmentation method is collision-induced dissociation (CID), by which ions are activated *via* collisions with a target gas at user-defined kinetic energies. This gives rise to fragment ions, whose nature as well as threshold energy can provide information regarding the stability and fragmentation pathway(s) of the precursor ion. Another powerful addition to mass spectrometry is ion mobility (together IM-MS), which separates ions based on their time to traverse a gas-filled drift cell. Here, structural information is provided in the form of collisional cross sections (CCS), which correspond to the size and shape of a given ion and can be compared to values computed from candidate geometries.

For the majority of MS and IM-MS experiments, neutral sample molecules, including all the complexes investigated in this work, require the addition of charge-carrying species (A^+^/A^–^) in order to be studied. The occurrence of different adduct ions [M + A]^+^ and [M + A]^−^ can be tuned by using A^+^/A^–^ containing solutions and/or by changing ion source conditions.^1^ These charge-carrying species can also affect intrinsic properties of the sample molecules, as previously reported for a range of compound classes such as carbohydrates,^2^ glycans and glycopeptides,^3^ steroids,^4^ fullerenes,^5^ and macrocycles.^5,6^ For example, Kellner *et al.* showed that the formation, disassembly, and stability of crown ether fullerene dimers depend on the size of the alkali metal cations used, which was attributed to different binding sites.^5^ Rister *et al.* used IM-MS to separate isobaric steroids and reported varying CCS resolutions for different A^+^, highlighting the relevance of choosing the most suitable charge-carrying species for this application.^4^

In the last decades, the interest in metallosupramolecular complexes has rapidly grown along with a simultaneous increase in the complexity of the synthesized molecules.^7−9^ We have been studying a family of polymetallic supramolecules that can be used as lithographic resists^10−12^ and have been proposed as qubits in quantum information processing.^13−16^ The structural characterization of these and other polymetallic complexes is often difficult, particularly when X-ray crystallography or reliable computations are not feasible because of the complex size.^17^ IM-MS has been applied to study and characterize polymetallic complexes in the past;^18−21^ however, systematic studies that examine the impact of fundamental building blocks on stability and structure are rare.

**Figure 1:**
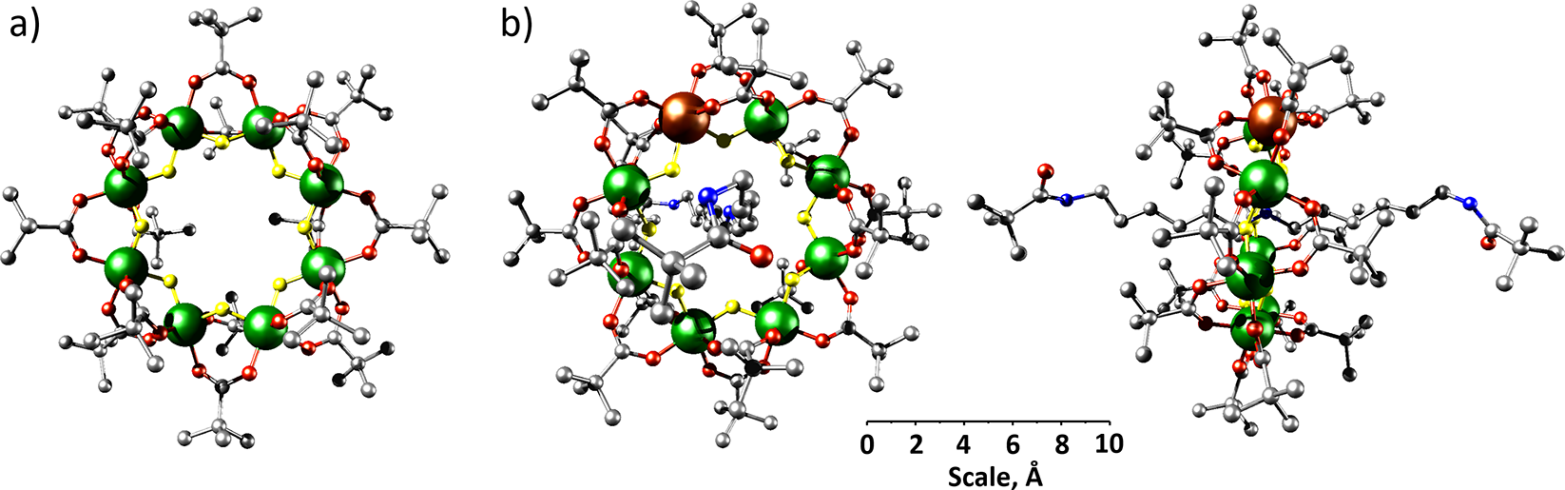
Structure of (a) the chromium-wheel complex **Ring_Cr_** (top view)^22^ and (b) the rotaxane **Am_Cu_** (top and side views)^23^ with a scale in Å (green, chromium; brown, copper; yellow, fluorine; red, oxygen; gray, carbon; blue, nitrogen). Hydrogen atoms are omitted for clarity.

We have recently used IM-MS to study the disassembly/fragmentation mechanisms and energetics of polymetallic rings and [2]-rotaxanes with the general formula [NH_2_RR′][Cr_7_MF_8_(O_2_C^*t*^Bu)_16_] (M = Mn^II^, Fe^II^, Co^II^, Ni^II^, Cu^II^, Zn^II^, and Cd^II^),^21^ as well as similar {Cr*_x_*Cu_2_} hourglass structures (*x* = 10, 12) and a {Cr_12_Gd_4_} complex.^36^ We showed how the d-metal composition, the R, R′ groups, and the general topology affect the stability and conformational landscape of these systems. Here, we extend this work and investigate the influence of different cations A^+^ (A^+^ = H^+^, Na^+^, K^+^, and Cs^+^), as well as H_2_O, on the disassembly, stability, and conformational landscape of metallosupramolecular complexes, namely, the homometallic ring [Cr_8_F_8_(O_2_C^*t*^Bu)_16_] = “**Ring_Cr_**” (Figure 1a) and the rotaxane families **Ph_M_** and **Am_M_**, [NH_2_RR′][Cr_7_MF_8_(O_2_C^*t*^Bu)_16_], where for **Ph_M_**, the thread [NH_2_RR′]^+^ is [NH_2_(CH_2_Ph)(CH_2_CH_2_Ph)]^+^ (“**TPh**^+^”) and for **Am_M_**, the thread [NH_2_RR′]^+^ is [NH_2_(C_6_H_12_NHC(O)^*t*^Bu)_2_]^+^ (“**TAm**^+^”), with M = Mn^II^, Fe^II^, Co^II^, Ni^II^, Cu^II^, Zn^II^, and Cd^II^ (Figure 1b for **Am_Cu_**). Our results show that the use of different charge carriers or small molecules can inform on structural trends in polymetallic complexes. Experimental and computational details can be found in the Supporting Information.

## Results and Discussion

### Encapsulating Charge Carriers and Small Molecules in **Ring_Cr_**

The neutral, homometallic wheel **Ring_Cr_** is a Cr_8_ octagon, in which each edge is bridged by one fluoride inside the ring and two pivalate ligands outside (O_2_C^*t*^Bu = Piv^–^; Figure 1a).^22^ In previous studies, we showed that **Ring_Cr_** can encapsulate both small neutral and cationic species, making this compound an ideal example to investigate the properties of different adducts using IM-MS.^22,25^**Ring_Cr_** was transferred to the gas phase from solutions of the iodides AI (A^+^ = Na^+^, K^+^, and Cs^+^; Figure S1 for NaI) or by adding formic acid. Various cationic adducts [**Ring_Cr_** + A]^+^ (A^+^ = Na^+^, Na^+^ + H_2_O, K^+^, and Cs^+^) were observed, as well as the oxidized, odd-numbered electron cation [**Ring_Cr_** + H_2_O]^+^. Figure S2 shows the isotopic distribution of the latter complex, which excludes an additional proton as the source of the positive charge.

**Figure 2:**
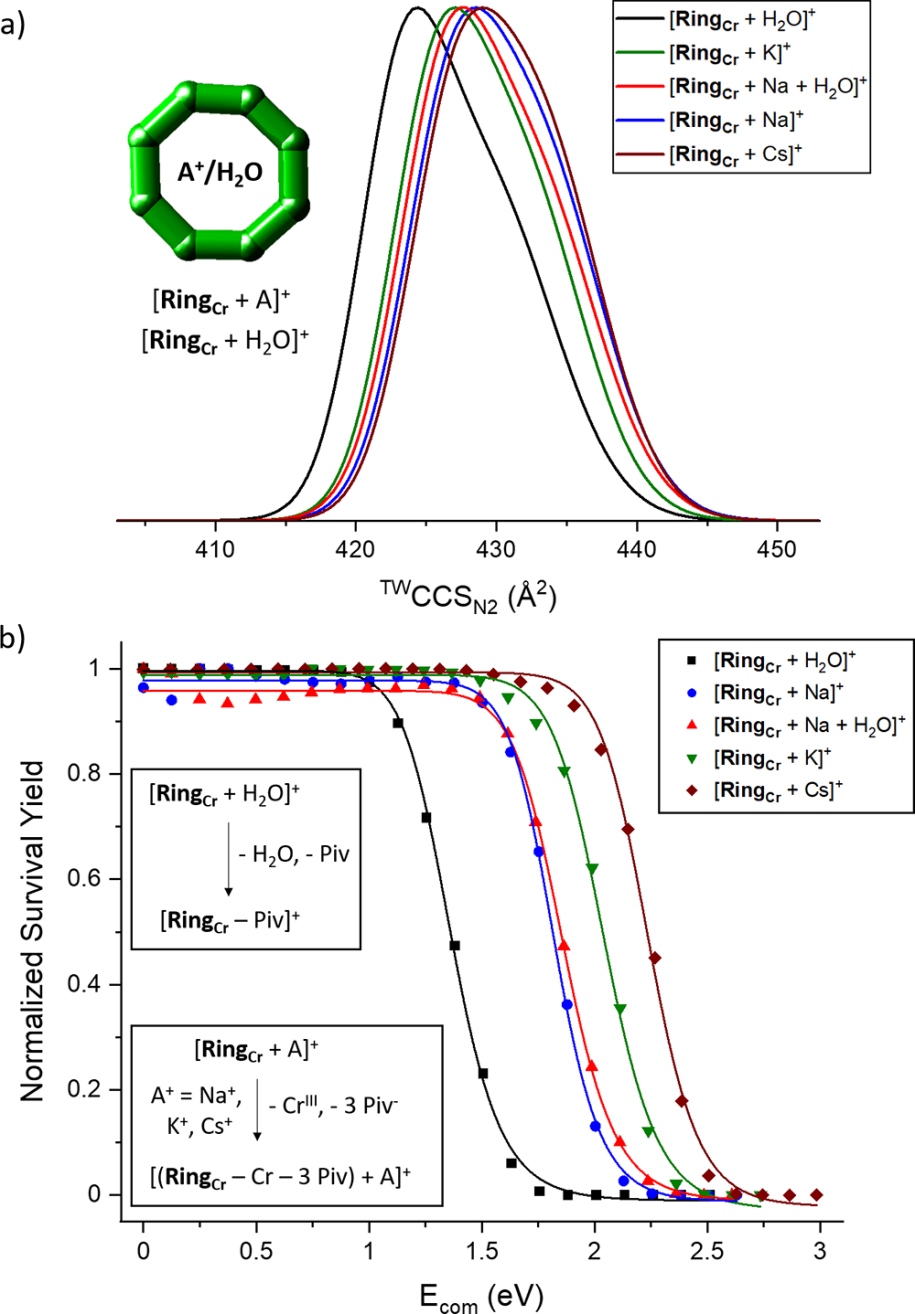
(a) ^TW^CCS_N2_ distributions and (b) survival yield plots for [**Ring_Cr_** + A]^+^ (A^+^ = Na^+^, Na^+^ + H_2_O, K^+^, and Cs^+^) and [**Ring_Cr_** + H_2_O]^+^. Inset: Schematic structure of [**Ring_Cr_** + A]^+^ and [**Ring_Cr_** + H_2_O]^+^. The main dissociation pathways of each adduct are presented. Separated plots with the corresponding standard deviations and two Gaussian fits can be found in Figure S3. Data were averaged over four datasets, except for [**Ring_Cr_** + H_2_O]^+^, for which two sets were used. The order of the ^TW^CCS_N2_ distributions presented here is H_2_O, K^+^, Na^+^/H_2_O, Na^+^, and Cs^+^ from lowest to highest ^TW^CCS_N2_ maximum.

The identity of the added species had no major impact on the conformational landscape of the adducts, as measured by IM-MS, and only small differences were observed in their CCS distributions determined by traveling wave ion mobility spectrometry in nitrogen gas (^TW^CCS_N2_; Figure 2a and Table 1). [**Ring_Cr_** + H_2_O]^+^ was found to be slightly smaller than the alkali metal adducts, and all species appear with a slightly asymmetric peak shape, presumably a result of two overlapping conformers, which could not be resolved further. The extent of the asymmetry varies slightly between the different adducts and from sample to sample (Figure S3), which suggests the occurrence of different, potentially interconverting ring adduct conformers, attributable to varying locations of A^+^ or H_2_O in **Ring_Cr_**. Theoretical ^TM^CCS_N2_ values were predicted for all five cations from their density functional theory (DFT)-optimized structures (Figures S4–S8) using the trajectory method (TM) of IMoS (Table S1).^26^ These were found to be ≈9% larger than the experiment, a difference that we previously observed and discussed in detail for the similar heterometallic rings [**Ring_M_**]^−^.^21^ In agreement with the experimental values, only minor differences in the calculated ^TM^CCS_N2_ values were found for different A^+^.

**Table 1:** *E*_50_ and ^TW^CCS_N2_ (TW = “Traveling Wave”) Values of the Cations [**Ring_Cr_** + A]^+^ (A^+^ = Na^+^, Na^+^ + H_2_O, K^+^, and Cs^+^) and [**Ring_Cr_** + H_2_O]^+^ Including Experimental Error^a^

cationic adduct	^TW^CCS_N2_ (Å^2^)	*E*_50_ (eV)
[**Ring_Cr_** + H_2_O]^+^	426.3 ± 1.0	1.364 ± 0.004
[**Ring_Cr_** + Na]^+^	430.0 ± 0.8	1.822 ± 0.004
[**Ring_Cr_** + Na + H_2_O]^+^	429.4 ± 0.4	1.860 ± 0.003
[**Ring_Cr_** + K]^+^	428.6 ± 1.1	2.045 ± 0.008
[**Ring_Cr_** + Cs]^+^	430.3 ± 0.9	2.239 ± 0.005

a The ^TW^CCS_N2_ values are found from the mid-point of the full width at half-maximum (FWHM) of the ^TW^CCS_N2_ distributions and were averaged over four datasets, except for [**Ring_Cr_** + H_2_O]^+^, for which two sets were averaged. Data for [**Ring_Cr_** + Na]^+^ and [**Ring_Cr_** + Na + H_2_O]^+^ could both represent the latter, if water is lost post the drift region.

In order to assess the stability of the different adducts, they were isolated, activated *via* collisions with nitrogen gas (CID), and the relative intensity of the precursor ions (“survival yield”) was derived for different center-of-mass energies *E*_com_ (Figure 2b including fragmentation pathways). The *E*_com_ at which the precursor ion decay reaches 50% (“*E*_50_”) is often employed as a relative measure of ion stability^27−29^ and was enumerated for the adducts of **Ring_Cr_**. Although all adducts present a similar conformational landscape, their stability significantly varies, with [**Ring_Cr_** + Cs]^+^ being the most stable species (Figure 2b and Table 1). A significantly lower *E*_50_ value was observed for the alkali metal cation [**Ring_Cr_** + K]^+^ and lower still for [**Ring_Cr_** + Na]^+^, showing that the stability trend correlates with the alkali metal size. To rationalize this behavior, DFT-optimized structures were generated for [**Ring_Cr_** + A]^+^ (A^+^ = Na^+^, K^+^, and Cs^+^). In these geometries, Cs^+^ is located in the center of the ring, whereas K^+^ and more strongly pronounced Na^+^ are closer to the edge of the interior (Figures S4–S6). The calculated structures are also in agreement with our previous findings from crystal structures, where Cs^+^ was shown to fill the cavity of the similar heterometallic [**Ring_Co_**]^−^ (discussed further below), binding to eight fluorides,^30^ whereas the smaller Na^+^ did not bind to all fluorides of [**Ring_Co_**]^−^.^31^ As the fragmentation channel of [**Ring_Cr_** + A]^+^ (A^+^ = Na^+^, K^+^, and Cs^+^) involves the disruption of several Cr–O (O from Piv^–^) and Cr–F bonds (Figure 2b), the stability of the entire complex presumably depends on the strength of these bonds. In the DFT-optimized structures, both the Cr–O and Cr–F bond length are on average in the order Cs^+^ < K^+^ < Na^+^ (Supplementary Dataset); however, the Cr–F bonds are always longer than in the neutral **Ring_Cr_** and are hence destabilized by A^+^. The trend in Cr–F bond lengths indicates that Cs^+^ has the least destabilizing effect on the ring structure, followed by K^+^ and Na^+^, which is in agreement with the experimental *E*_50_ trend. For [**Ring_Cr_** + A]^+^ (A^+^ = Na^+^ and K^+^), we also found shorter and hence more stable Cr–O and Cr–F bonds for those close to A^+^, suggesting that Na^+^ and K^+^ stabilize the region of the complex where they bind (Supplementary Dataset).

[**Ring_Cr_** + Na]^+^ was observed in the mass spectrum of **Ring_Cr_** in a solution of NaI but could not be isolated in the quadrupole mass filter (Figures S1 and S2). However, the MS^2^ spectrum of the sodiated water adduct [**Ring_Cr_** + Na + H_2_O]^+^ yielded both product ions [**Ring_Cr_** + A]^+^ (A^+^ = Na^+^ and Na^+^ + H_2_O) without further collisional activation (Figure S9), indicating a small energy barrier for the loss of water. This was confirmed by highly similar *E*_50_ values of [**Ring_Cr_** + Na]^+^ and [**Ring_Cr_** + Na + H_2_O]^+^, although the latter was found to be slightly more stable (Figure 2b and Table 1), consistent with stable inclusion of water.

Different disassembly channels were observed for the species [**Ring_Cr_** + A]^+^ (A^+^ = Na^+^, Na^+^ + H_2_O, K^+^, and Cs^+^) and the oxidized, odd-numbered electron cation [**Ring_Cr_** + H_2_O]^+^ (Figure 2b). While the alkali metal species show the loss of one chromium center and three anionic ligands (predominantly three pivalates; Figure S10a), [**Ring_Cr_** + H_2_O]^+^ fragments predominantly *via* the loss of water and one likely neutral pivalate (see discussion below, Figure S10b). [**Ring_Cr_** + H_2_O]^+^ was found to have the smallest *E*_50_ value of all studied adducts, which we attribute to a thermodynamically favorable reduction during fragmentation. The DFT-optimized structure of [**Ring_Cr_** + H_2_O]^+^ (Figure S7) was used to investigate which part of this unusual cation is oxidized. We performed a natural orbital analysis together with the Merz–Kollman and Mulliken charges, as well as the corresponding spin densities of the latter (Supplementary Dataset). The positive charge appears to be somewhat delocalized, but the two pivalates bridging Cr–F–Cr on the opposite site of the water binding are notably more positive and with more radical character than other pivalates. All Cr^III^ centers seem to be maintained.

### Stability of the Rotaxane Ions [**Am_M_** + A]^+^ and [**Ph_M_** + A]^+^ (A^+^ = Na^+^, K^+^, and Cs^+^)

**Figure 3:**
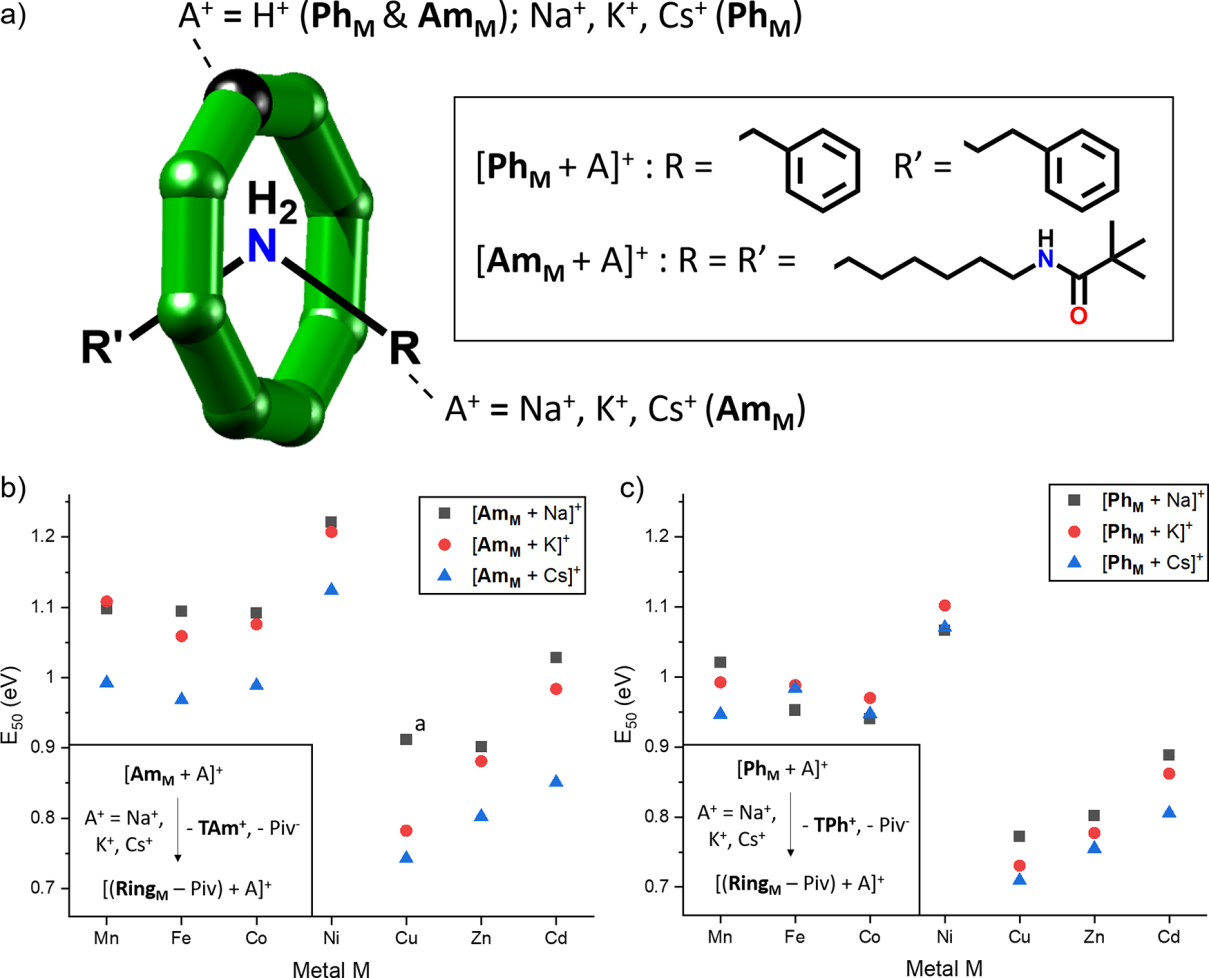
(a) Schematic structure of [**Ph_M_** + A]^+^ and [**Am_M_** + A]^+^ including presumed locations of A^+^. *E*_50_ values of (b) [**Am_M_** + A]^+^ and (c) [**Ph_M_** + A]^+^ (A^+^ = Na^+^, K^+^, and Cs^+^; M = Mn^II^, Fe^II^, Co^II^, Ni^II^, Cu^II^, Zn^II^, and Cd^II^) with respect to M. The main dissociation reactions are presented. ^a^For [**Am_Cu_** + Na]^+^, the main dissociation pathway is the loss of Cu^II^ and two Piv^–^. Error bars are shown but, in all cases, are smaller than the symbol size. Data for the series [**Ph_M_** + Na]^+^ were obtained from our previous work.^21^

The rotaxane families **Am_M_** and **Ph_M_** (Figure 3a) involve a secondary ammonium cation [NH_2_RR′]^+^ (“thread”), surrounded by a heterometallic ring [**Ring_M_**]^−^ (M = Mn^II^, Fe^II^, Co^II^, Ni^II^, Cu^II^, Zn^II^, and Cd^II^). These rings are similar to **Ring_Cr_**, but with one Cr^III^ center exchanged for a divalent metal M^II^, leading to an overall negative charge.^24,32^ Previously, we have extensively studied the disassembly of [**Ring_M_**]^−^, [**Am_M_** + A]^+^, and [**Ph_M_** + A]^+^ (A^+^ = H^+^ and Na^+^) using IM-MS, and demonstrated that the divalent metal M and the thread strongly influence the *E*_50_ value and disassembly mechanism of these cations.^21^ Major differences were also observed between protonated and sodiated adducts, showing that the charge-carrying species is an important factor when considering the disassembly of these complexes (Table 2 and Figure S11). Here, we extend this work by enumerating the *E*_50_ values of the potassium and cesium adducts, [**Am_M_** + A]^+^ and [**Ph_M_** + A]^+^ (A^+^ = K^+^ and Cs^+^), and compare them to the data of the sodiated adduct ions (Table 2 and Figure 3b,c including dissociation reactions).^21^ The data show notable differences with respect to the alkali metal.

**Table 2:** *E*_50_ Values of [**Ph_M_** + A]^+^ and [**Am_M_** + A]^+^ (A^+^ = H^+^, Na^+^, K^+^, and Cs^+^) Including Experimental Error^a^

*E*_50_ (eV)	Mn^II^	Fe^II^	Co^II^	Ni^II^	Cu^II^	Zn^II^	Cd^II^
[**Ph_M_** + H]^+^	0.283 ± 0.003		0.288 ± 0.005	0.331 ± 0.003	0.478 ± 0.014	0.377 ± 0.008	0.242 ± 0.002
[**Ph_M_** + Na]^+^	1.021 ± 0.001	0.953 ± 0.001	0.940 ± 0.002	1.066 ± 0.002	0.772 ± 0.002	0.802 ± 0.002	0.889 ± 0.002
[**Ph_M_** + K]^+^	0.992 ± 0.003	0.988 ± 0.004	0.970 ± 0.003	1.102 ± 0.004	0.731 ± 0.003	0.777 ± 0.002	0.862 ± 0.004
[**Ph_M_** + Cs]^+^	0.946 ± 0.002	0.984 ± 0.003	0.947 ± 0.003	1.071 ± 0.004	0.709 ± 0.002	0.755 ± 0.002	0.805 ± 0.004
[**Am_M_** + H]^+^	0.248 ± 0.003		0.256 ± 0.002	0.209 ± 0.002	0.450 ± 0.004	0.336 ± 0.002	0.218 ± 0.003
[**Am_M_** + Na]^+^	1.098 ± 0.005	1.094 ± 0.002	1.092 ± 0.005	1.221 ± 0.003	0.912 ± 0.004	0.902 ± 0.003	1.028 ± 0.007
[**Am_M_** + K]^+^	1.108 ± 0.004	1.059 ± 0.003	1.076 ± 0.003	1.207 ± 0.004	0.782 ± 0.007	0.881 ± 0.003	0.984 ± 0.003
[**Am_M_** + Cs]^+^	0.993 ± 0.003	0.968 ± 0.003	0.989 ± 0.003	1.124 ± 0.003	0.743 ± 0.003	0.802 ± 0.003	0.851 ± 0.003

a Data for [**Ph_M_** + A]^+^ and [**Am_M_** + A]^+^ (A^+^ = H^+^ and Na^+^) were obtained from our previous work.^21^

For six of the seven divalent metals studied, *E*_50_ values for [**Am_M_** + A]^+^ (A^+^ = Na^+^, K^+^, and Cs^+^) each show the Na^+^ adducts to be most stable, followed by the respective K^+^ and Cs^+^ species; the minor exception is for M = Mn^II^ (Figure 3b). However, for the rotaxane adducts [**Ph_M_** + A]^+^ (A^+^ = Na^+^, K^+^, and Cs^+^), the alkali metal trend varies with the divalent metal M. The stability order is Na^+^ > K^+^ > Cs^+^ for M = Mn^II^, Cu^II^, Zn^II^, and Cd^II^ (Figure 3c). In contrast for M = Fe^II^, Co^II^, and Ni^II^, the potassium adducts are most stable, followed by [**Ph_M_** + Cs]^+^ and [**Ph_M_** + Na]^+^.

The observed trends can be rationalized with the binding site of the alkali metals. For **Am_M_**, the alkali metal cation A^+^ likely coordinates at the end groups of **TAm^+^**, whose amides are well-known to bind alkali metals effectively in the gas phase (bond dissociation energies: 120–160 kJ mol^–1^; Figure 3a).^33^ Less likely is binding to the *tert*-butyl groups outside the ring, which is energetically disfavored compared to the **TAm^+^** end groups,^33^ or to the heteroatoms of the anionic ligands, for which crystal structures suggest insufficient space for the alkali metals to coordinate (Figure 1b for **Am_Cu_**).^23^ For **TPh^+^**, the binding of A^+^ at the phenyl end groups is less favored compared to **TAm^+^** (bond dissociation energies: 65–110 kJ mol^–1^),^33^ and here, crystal structures also indicate that the end groups are too close to the ring to allow coordination of A^+^ without major distortions.^21^ This presumably leaves the outside of the ring as the only possible location of A^+^ in [**Ph_M_** + A]^+^ (A^+^ = Na^+^, K^+^, and Cs^+^; Figure 3a), likely close to the pivalates that are bound to M^II^, as the charge density there will be lower than at the ligands exclusively bound to Cr^III^.

The disassembly mechanisms of [**Am_M_** + A]^+^ and [**Ph_M_** + A]^+^ likely involve the release of the thread *via* slipping through the ring (and the loss of an anionic pivalate); however, the data indicate that A^+^ remains on the product [(**Ring_M_** – Piv) + A]^+^ after fragmentation.^21^ For [**Am_M_** + A]^+^, we suggest an alkali metal transfer from the thread to the ring during thread loss (Figure S12), which leads to the coordination of A^+^ in the center of the ring, similar to the alkali metal adducts of **Ring_Cr_** (Figure 2) and our previously reported, neutral bulk-phase complexes [**Ring_Co_** + A] (A^+^ = Na^+^, Rb^+^, and Cs^+^).^30,31^ Na^+^ is the smallest of the three studied alkali metal cations, with the highest charge density, leading to stronger bonds with the thread end groups than for K^+^ and even more than for Cs^+^.^33^ Additionally, the disassembly products [(**Ring_M_** – Piv) + A]^+^ are similar to [**Ring_Cr_** + A]^+^ and presumably also most stabilized in the order Cs^+^ > K^+^ > Na^+^ (Table 1 and Figure 2b). Both trends would lead to the loss of the thread and transfer of A^+^ to the ring being most favored for Cs^+^ and least favored for Na^+^, which results in the highest *E*_50_ values for Na^+^ as observed for almost all [**Am_M_** + A]^+^.

For [**Ph_M_** + A]^+^, where A^+^ is likely located outside of the ring and bound to pivalates that are coordinated to M, we observed the same trend for the divalent metals with larger covalent radii^34^ (Cd^II^, Mn^II^, Cu^II^, and Zn^II^; Figure 3c) as for [**Am_M_** + A]^+^. This could be explained with the better fit of the smaller A^+^ into the space between the *tert*-butyl groups, resulting in stronger bonds between A^+^ and the pivalate ligands in the order Na^+^ > K^+^ > Cs^+^. These interactions presumably stabilize the entire region of the complex, similar to the alkali metal adducts [**Ring_Cr_** + A]^+^ discussed above.

Apart from the nominal stability of the precursor ion, the product ion stability also has an impact on the *E*_50_ value, as more stable products decrease the *E*_50_ value of the precursor ion. This can be used to rationalize the more unusual trends for the [**Ph_M_** + A]^+^ species with smaller covalent radii^34^ of M (Fe^II^, Co^II^, and Ni^II^), which follow the *E*_50_ trend K^+^ > Cs^+^ > Na^+^ (Figure 3c). Because of the reduced space at the outside of the ring due to smaller M, A^+^ presumably cannot bind as closely to the pivalates bound to M and the precursor ion stability is likely a less significant factor for the determination of *E*_50_. However, the product ions [(**Ring_M_** – Piv) + A]^+^ are more spacious after the loss of the thread and one anionic pivalate ligand. Assuming that A^+^ remains outside the ring, these product species are likely stabilized in the order Na^+^ > K^+^ > Cs^+^, which would lead to the reverse trend for the *E*_50_ values of the precursor ions [**Ph_M_** + A]^+^ (Cs^+^ > K^+^ > Na^+^). We suggest that the experimental trend (K^+^ > Cs^+^ > Na^+^) is an overlap of the two trends of precursor and product ion stability, which is shifted toward product ions for [**Ph_M_** + A]^+^ with smaller M. Although these considerations were rationalized using covalent radii, the use of ionic radii of M yielded a similar but slightly worse correlation.^35^

### Conformational Flexibility of the Rotaxane Ions [**Am_M_** + A]^+^ and [**Ph_M_** + A]^+^ (A^+^ = H^+^ and Na^+^)

**Figure 4:**
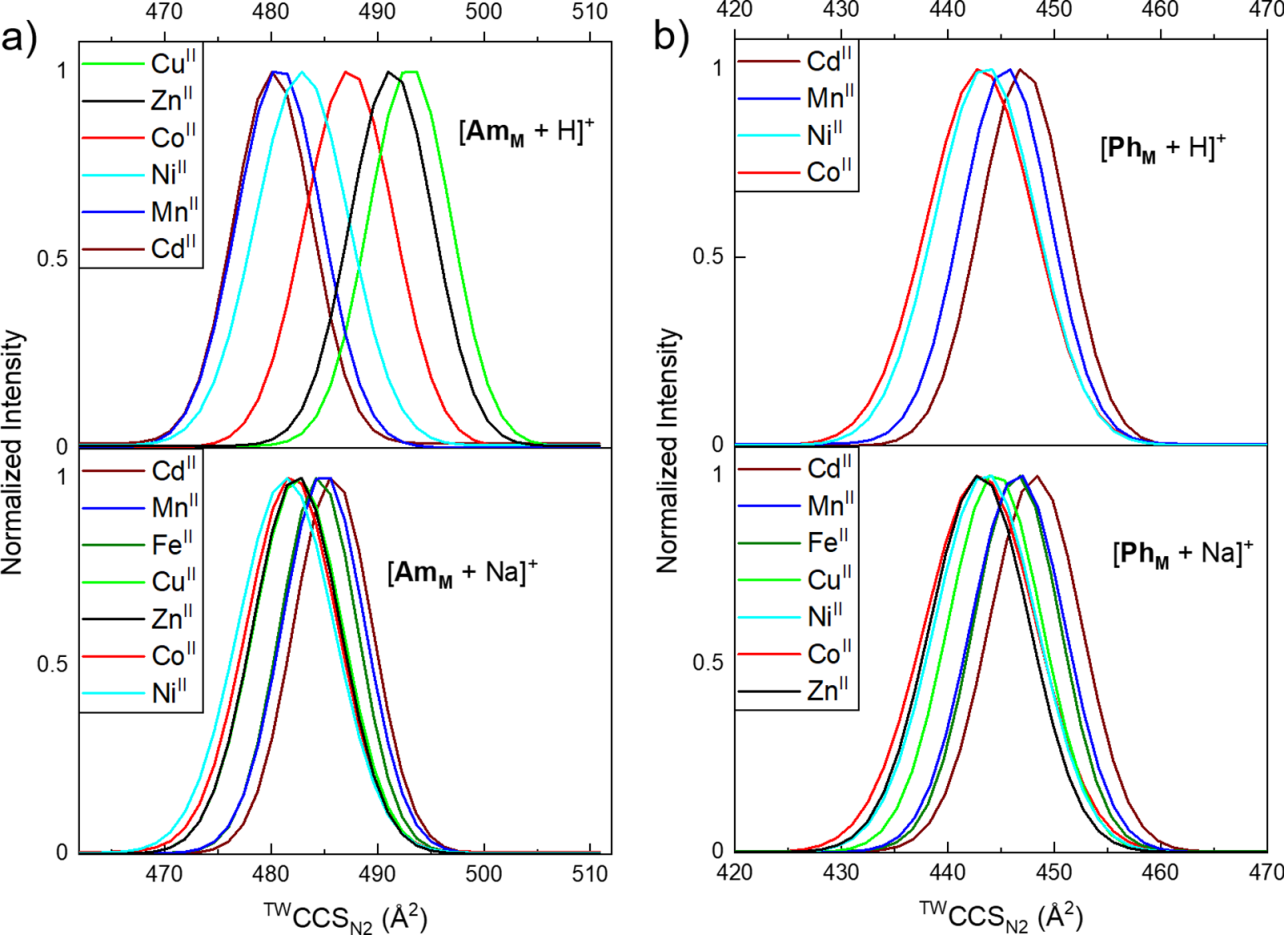
^TW^CCS_N2_ distributions of (a) [**Am_M_** + A]^+^ and (b) [**Ph_M_** + A]^+^ (A^+^ = H^+^ and Na^+^). One dataset is shown for each distribution and all experimental errors of ^TW^CCS_N2_ are smaller than 0.5% (Table S2). For each plot, legends are arranged from highest to lowest ^TW^CCS_N2_ maximum, as presented in the figure. The distribution for [**Am_Fe_** + H]^+^ and [**Ph_M_** + H]^+^ (M = Fe^II^, Cu^II^, and Zn^II^) could not be obtained because of overlapping signals.

The conformational landscape of the studied rotaxanes can also depend on A^+^ and this effect was most prominently shown for [**Am_M_** + Na]^+^ and [**Am_M_** + H]^+^ (Figure 4a and Table S2). The series of sodiated species shows only minor differences in ^TW^CCS_N2_ (Δ^TW^CCS_N2_ = 5.7 Å^2^), whereas the distributions of [**Am_M_** + H]^+^ are spread over a much broader range (Δ^TW^CCS_N2_ = 12.9 Å^2^). Here, the complexes of M = Cd^II^, Mn^II^, and Ni^II^ are smaller than the one with M = Co^II^, which is smaller than the species with M = Zn^II^ and Cu^II^. In contrast, this larger spread was neither observed for [**Ph_M_** + H]^+^ (Δ^TW^CCS_N2_ = 3.3 Å^2^) nor [**Ph_M_** + Na]^+^ (Δ^TW^CCS_N2_ = 4.5 Å^2^; Figure 4b and Table S2).

The variation in the ^TW^CCS_N2_ data of [**Am_M_** + H]^+^ can again be rationalized with the binding site of H^+^ and the covalent radii of M. As shown in our previous work,^21^ the protonated species [**Am_M_** + H]^+^ and [**Ph_M_** + H]^+^ dissociate *via* the loss of pivalic acid (HPiv), indicating that the proton is located at the ring, and likely bound to those Piv^–^ that are bound to M^II^ because of the lower charge density there compared to the Cr^III^ sites. For the complexes with the largest d-metals, Cd^II^ and Mn^II^, protons presumably are located between the anionic pivalate ligands, coordinated to the −O and possibly −F atoms. Here, H^+^ could draw the complex together leading to a more compact conformation. Conversely, the smaller metals (M = Co^II^, Cu^II^, and Zn^II^) might not provide enough inter-ligand space, making pivalate protonation only possible at the outside of the ring. This could result in an extended ring conformation with higher ^TW^CCS_N2_ (Figure 4a top), in which the proton pulls the ligands toward the ring exterior. The M = Ni^II^ species appears more compact than the Co^II^ compound and is difficult to rationalize.

The absence of this spread for the sodiated ions [**Am_M_** + Na]^+^ and [**Ph_M_** + Na]^+^ (Figure 4) can be explained by the different binding sites of Na^+^, as discussed above, and the lower charge density of Na^+^, which possibly minimizes the distortions induced by A^+^. The different behavior of [**Ph_M_** + H]^+^ (Figure 4b top) is presumably a result of the proximity of the phenyl end groups, limiting the binding sites of H^+^ at the ring and hence the conformational flexibility of [**Ph_M_** + H]^+^.

We quantified the FWHM of the ^TW^CCS_N2_ distribution for each rotaxane ion [**Am_M_** + A]^+^ and [**Ph_M_** + A]^+^ (A^+^ = H^+^ and Na^+^) as a measure of their conformational flexibility (Table S3). Significant differences in FWHM (^TW^CCS_N2_) were observed, although the ions involving the large metal Cd^II^ and to a lesser extent Mn^II^ yield in most cases narrower distributions than the related species with smaller M. This suggests more rigidity for the ions with Cd^II^ and Mn^II^ possibly because of an enlarged and asymmetric ring shape. Comparison of the alkali metal adducts for the two selected ions [**Ph_Mn_** + A]^+^ and [**Am_Co_** + A]^+^ (A^+^ = Na^+^, K^+^, and Cs^+^) shows that both ^TW^CCS_N2_ and FWHM (^TW^CCS_N2_) increase as the size of the alkali metal cations increases (Cs^+^ > K^+^ > Na^+^; Tables S2 and S3). This can be explained with the larger size of A^+^ and hence weaker bonding, which slightly enhances the size and flexibility of the entire rotaxane.

## Conclusions

In conclusion, we demonstrate how the variation of adduct ions can be used to investigate molecular properties of metallosupramolecular complexes using IM-MS and CID. By investigating the stabilities and conformational flexibilities of a series of polymetallic rings and rotaxanes, different structural trends were derived and shown to correlate with crystal structure data. Further comparison with the DFT-optimized structures of the heterometallic [**Ring_M_**]^−^ yields a similar trend of the averaged metal–metal distances with varying M (largest for Cd^II^ and Mn^II^),^21^ which suggests good agreement in evaluating transition metal properties between crystallographic data, DFT calculations, and the here presented adduct ion studies. The ability of the latter to provide trends for structure and stability is particularly attractive for investigations of larger metallosupramolecular complexes, for which X-ray crystallography and DFT may often not be feasible.^17^

## Supporting Information Available

Experimental and computational details; mass spectra, tandem mass spectra, ion mobility data, DFT-optimized structures and ^TM^CCS_N2_ values of **Ring_Cr_** adducts; E_50_, ^TW^CCS_N2_, and FWHM (^TW^CCS_N2_) values of [**Am_M_** + A]^+^ and [**Ph_M_** + A]^+^; suggested disassembly mechanism of [**Am_M_** + A]^+^ (A^+^ = Na^+^, K^+^, and Cs^+^) (PDF)

## Notes

Supporting data referred to in this manuscript is contained within a supplementary information document and in a supplementary dataset available on Figshare 10.6084/m9.figshare.21333786. The latter contains the raw data of ion mobility mass spectrometry and mass spectrometry measurements as well as the outputs from DFT calculations.
