# Supplementary material for: Adduct Ions as Diagnostic Probes of Metallosupramolecular Complexes Using Ion Mobility Mass Spectrometry
Source: Inorg Chem. 2023 Jan 30;62(6):2672–9. doi: 10.1021/acs.inorgchem.2c03698 (PMC9930111; doi:10.1021/acs.inorgchem.2c03698)
Supplement: Supplementary file 1 — ic2c03698_si_001.pdf [file ic2c03698_si_001.pdf]

Supporting Information

Adduct Ions as Diagnostic Probes of Metallosupramolecular Complexes using Ion Mobility Mass Spectrometry

Niklas Geue[1], Tom S. Bennett[2], Lennart A. I. Ramakers[1], Grigore A. Timco[2], Eric. J. L. McInnes[2], Neil A. Burton[2], P. B. Armentrout[3], Richard E. P. Winpenny[2] and Perdita E. Barran[1,*]

[1]Michael Barber Centre for Collaborative Mass Spesctrometry, Manchester Institute of Biotechnology, Department of Chemistry, The University of Manchester, 131 Princess Street, Manchester, M1 7DN, UK. [2]Department of Chemistry, The University of Manchester, Oxford Road, Manchester, M13 9PL, UK. [3]Department of Chemistry, University of Utah, Salt Lake City, Utah 84112, USA.

[*]Corresponding Author: perdita.barran@manchester.ac.uk

Table of Contents

Experimental and Computational Details 3

Figure S1: Mass spectrum of **Ring$_{Cr}$** in NaI. 5

Figure S2: Predicted (top) and observed (bottom) isotopic distribution of [**Ring$_{Cr}$** + H_2O]$^+$ 6

Figure S3: $^{TW}CCS_{N2}$ distributions of [**Ring$_{Cr}$** + A]$^+$ (A$^+$ = Na$^+$, Na$^+$ + H_2O, K$^+$, Cs$^+$) and [**Ring$_{Cr}$** + H_2O]$^+$ including experimental error 7

Figure S4: DFT optimised structure of [**Ring$_{Cr}$** + Cs]$^+$ 8

Figure S5: DFT optimised structure of [**Ring$_{Cr}$** + K]$^+$ 9

Figure S6: DFT optimised structure of [**Ring$_{Cr}$** + Na]$^+$ 10

Figure S7: DFT optimised structure of [**Ring$_{Cr}$** + H_2O]$^+$ 11

Figure S8: DFT optimised structure of [**Ring$_{Cr}$** + Na + H_2O]$^+$ 12

Table S1: $^{TM}CCS_{N2}$ and $^{TW}CCS_{N2}$ values of [**Ring$_{Cr}$** + A]$^+$ (A$^+$ = Na$^+$, Na$^+$ + H_2O, K$^+$, Cs$^+$) and [**Ring$_{Cr}$** + H_2O]$^+$ 13

Figure S9: MS^2 spectrum of [**Ring$_{Cr}$** + Na + H_2O]$^+$ at E_{lab} = 0 eV 14

Figure S10: MS^2 spectra of a) [**Ring$_{Cr}$** + Cs]$^+$ at E_{lab} = 190 eV and b) [**Ring$_{Cr}$** + H_2O]$^+$ at E_{lab} = 110 eV. 15

Figure S11: E_{50} values of [**Ph$_M$** + A]$^+$ and [**Am$_M$** + A]$^+$ (A$^+$ = H$^+$, Na$^+$) 16

Figure S12: Suggested disassembly mechanism of [**Am$_M$** + A]$^+$ (A = Na$^+$, K$^+$, Cs$^+$) 17

Table S2: $^{TW}CCS_{N2}$ values of [**Am$_M$** + A]$^+$ and [**Ph$_M$** + A]$^+$ (A$^+$ = H$^+$, Na$^+$) 18

Table S3: *FWHM* ($^{TW}CCS_{N2}$) values of [**Am$_M$** + A]$^+$ and [**Ph$_M$** + A]$^+$ (A$^+$ = H$^+$, Na$^+$) 19

References 20

Experimental and Computational Details

Synthesis. **Ring$_{Cr}$**[1], as well as the rotaxane families **Am$_M$**[2] and **Ph$_M$**[3] (M = Mn^{II}, Fe^{II}, Co^{II}, Ni^{II}, Cu^{II}, Zn^{II}, and Cd^{II}) were synthesized using methods previously published by our groups. All reagents and solvents were purchased from Fluorochem, Alfa, Sigma-Aldrich or Fisher Scientific and used without further purification.

Sample Preparation. Samples were typically prepared in 4:1 toluene/methanol (**Ph$_M$, Ring$_{Cr}$**) or 7:3 methanol/toluene (**Am$_M$**), respectively, and 500 µM AI (A^+ = Na^+, K^+, Cs^+) were added. Final concentrations of 2 µM (Q Exactive UHMR) and 10 µM (Cyclic) were typically used. When necessary, 0.5% - 1% formic acid was added to enhance the signal of the ions [**Ph$_M$** + H]$^+$, [**Am$_M$** + H]$^+$ and [**Ring$_{Cr}$** + H_2O]$^+$.

nano-Electrospray Ionisation (nESI). All samples were transferred to the gas phase with a nESI source and were sprayed from borosilicate glass capillaries (World Precision Instruments, Stevenage, UK). Glass capillaries were pulled on the Flaming/Brown P-2000 laser puller (Sutter Instrument Company, Novato, CA, US). The capillary voltage (1.0 - 1.5 kV) was applied through a platinum wire (Diameter 0.125 mm, Goodfellow, Huntingdon, UK) inserted into the nESI capillaries. The source temperature was set to T = 23 °C (Cyclic) or T = 30 °C (Q Exactive UHMR).

Tandem Mass Spectrometry (MS^2). The Q Exactive Ultra-High-Mass-Range (UHMR) Hybrid Quadrupole-Orbitrap Mass Spectrometer (Thermo Fisher) was used for the calculation of all E_{50} values *via* tandem mass spectrometry experiments (MS^2) involving collision-induced dissociation (CID).[4] Target ions were *m/z*-isolated in a quadrupole filter, accelerated to a user-defined energy (E_{lab}: 0 - 300 eV) and injected into the higher-energy C-trap dissociation (HCD) cell, which contained nitrogen gas (trapping gas pressure parameter: 2.0). Fragment ions as well as non-fragmented precursor ions, were transferred to the Orbitrap mass analyser (resolution: 25000, AGC target: 3E6 ions, maximum inject time: 100 ms).

Ion mobility mass spectrometry (IM-MS) experiments were performed on a Select Series Cyclic IMS (Waters).[5] Following ionization (Cone Voltage: 20 - 60 V, Source Offset: 10 - 20 V, Purge Gas: 50 – 300 L/h), ions were injected into the cyclic ion mobility drift ring. In this region, ions were separated by using a non-uniform electric field under a constant nitrogen pressure with

travelling waves (TW, Height: 20 V), which push the ions through the drift region. Ions travelled one pass in the cyclic drift ring ("single path", separation time: 2 ms) and were then transferred (Transfer Energy: 4 – 15 V) to a time-of-flight mass analyser.

Data Processing. E_{50} values were obtained using the workflow described in our previous work.[3] Briefly, mass spectra were recorded at different collision energies and the share of the precursor ion count relative to the total ion count ("survival yield") was plotted vs. the collisional energy in the centre-of-mass frame (E_{com}, Figure 1a). Survival yield plots were fitted with a sigmoidal Hill function (Hill1 function in OriginPro 2020b), yielding the point (E_{50}) at which the survival yield reaches 0.5 or 50%. This E_{50} value is known as a relative measure of precursor ion stability in the gas phase. In some cases, contaminating species overlapped with the precursor ion, however their shares in the survival yield plots were subtracted before fitting.

Experimentally obtained arrival time distributions (ATD) were converted to collisional cross sections $^{TW}CCS_{N2}$ (TW = "Traveling Wave") via published calibration procedures[6] using the Agilent tune mix.[7]

Density Functional Theory. DFT calculations were carried out with Gaussian 16[8] utilizing the B3LYP exchange-correlation functional with the Grimme D3 empirical dispersion correction.[9] An effective core potential and its associated split valence basis set were used for transition and alkali metals (LANL2DZ),[10] and a 6-31G(d) basis set on other atoms. All structures were optimized to the default convergence criteria (RMS force < $3 \cdot 10^{-4}$ E_h/a_0). Metal electronic states were high spin, as found experimentally, with low deviations from $\langle S^2 \rangle$, although were ferromagnetically coupled. Atomic charges were obtained for the optimized structures at the same DFT level using the Merz-Kollman method with UFF based radii, and natural population analysis[11] was performed on [**Ring**$_{Cr}$ + H_2O]$^+$, as implemented in Gaussian 16.

Theoretical Collision Cross Section values ($^{TM}CCS_{N2}$, TM = "Trajectory Method") were obtained from the software IMoS by using the trajectory method in nitrogen gas including quadrupole potential (number of orientations: 3, gas molecules per orientation: 300,000, temperature: 298 K, pressure: 101,325 Pa = 1 atm).[12]

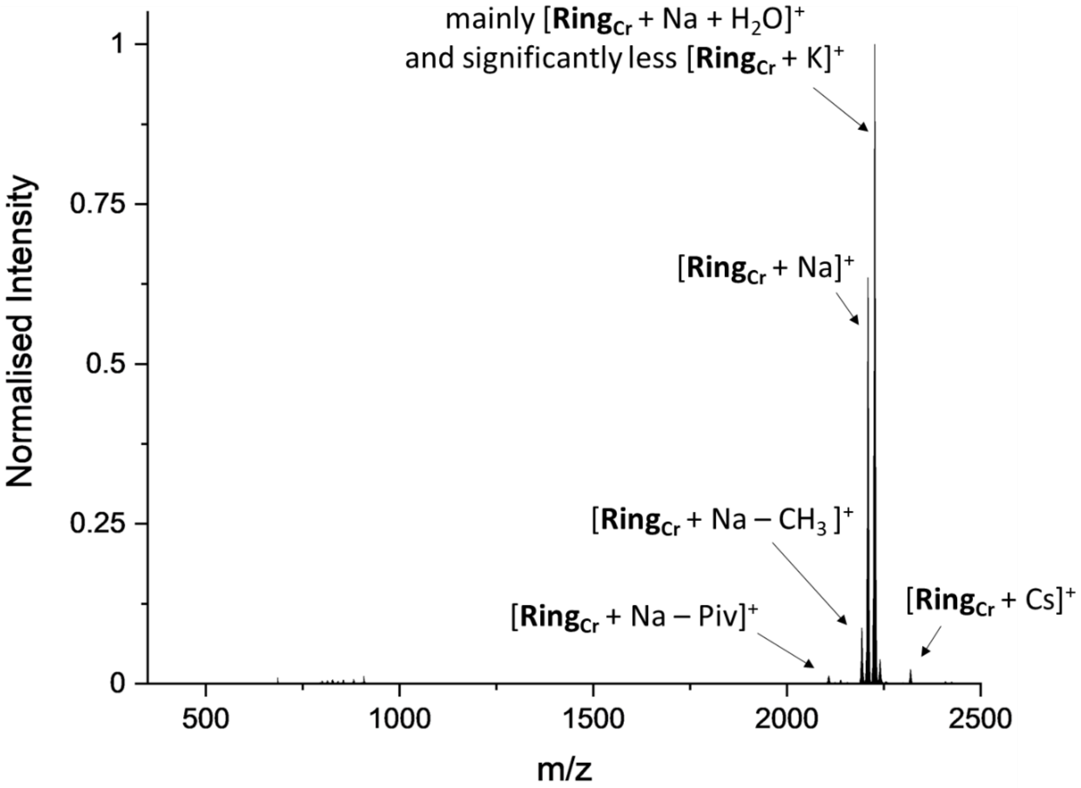

Figure S1: Mass spectrum of **Ring$_{\mathbf{Cr}}$** in NaI. 2 µM **Ring$_{\mathbf{Cr}}$** were used in 4:1 toluene/methanol and 500 µM NaI. Apart from the adducts [**Ring$_{\mathbf{Cr}}$** + A]$^+$ (A^+ = Na^+, Na^+ + H_2O, K^+, Cs^+), the metastable loss of one methyl group and the loss of a single, likely neutral pivalate ligand were observed from [**Ring$_{\mathbf{Cr}}$** + Na]$^+$.

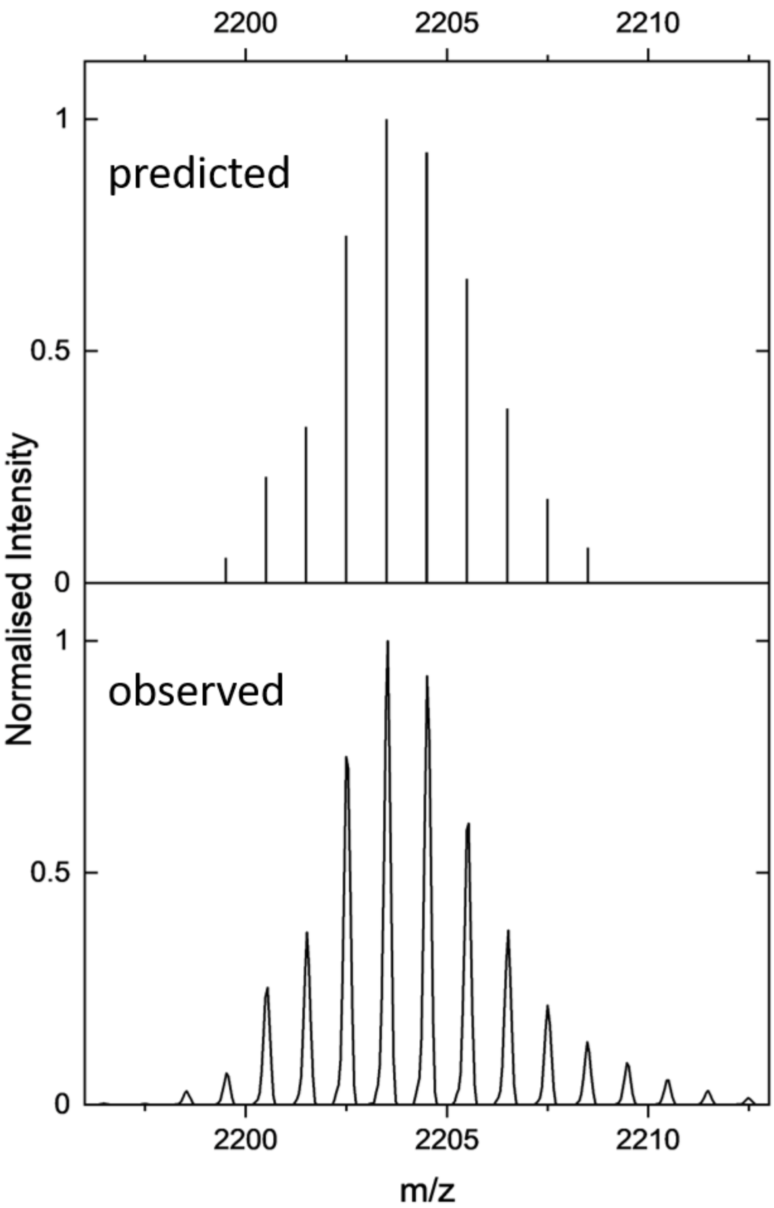

Figure S2: Predicted (top) and observed (bottom) isotopic distribution of [**Ring**$_{\mathbf{Cr}}$ + H_2O]$^+$ at *m/z* = 2203. 2 µM **Ring**$_{\mathbf{Cr}}$ were used in 4:1 toluene/methanol and 500 µM NaI. The range between *m/z* = 2200 and *m/z* = 2218 was isolated in the quadrupole mass filter to minimise the selection of [**Ring**$_{\mathbf{Cr}}$ + Na]$^+$.

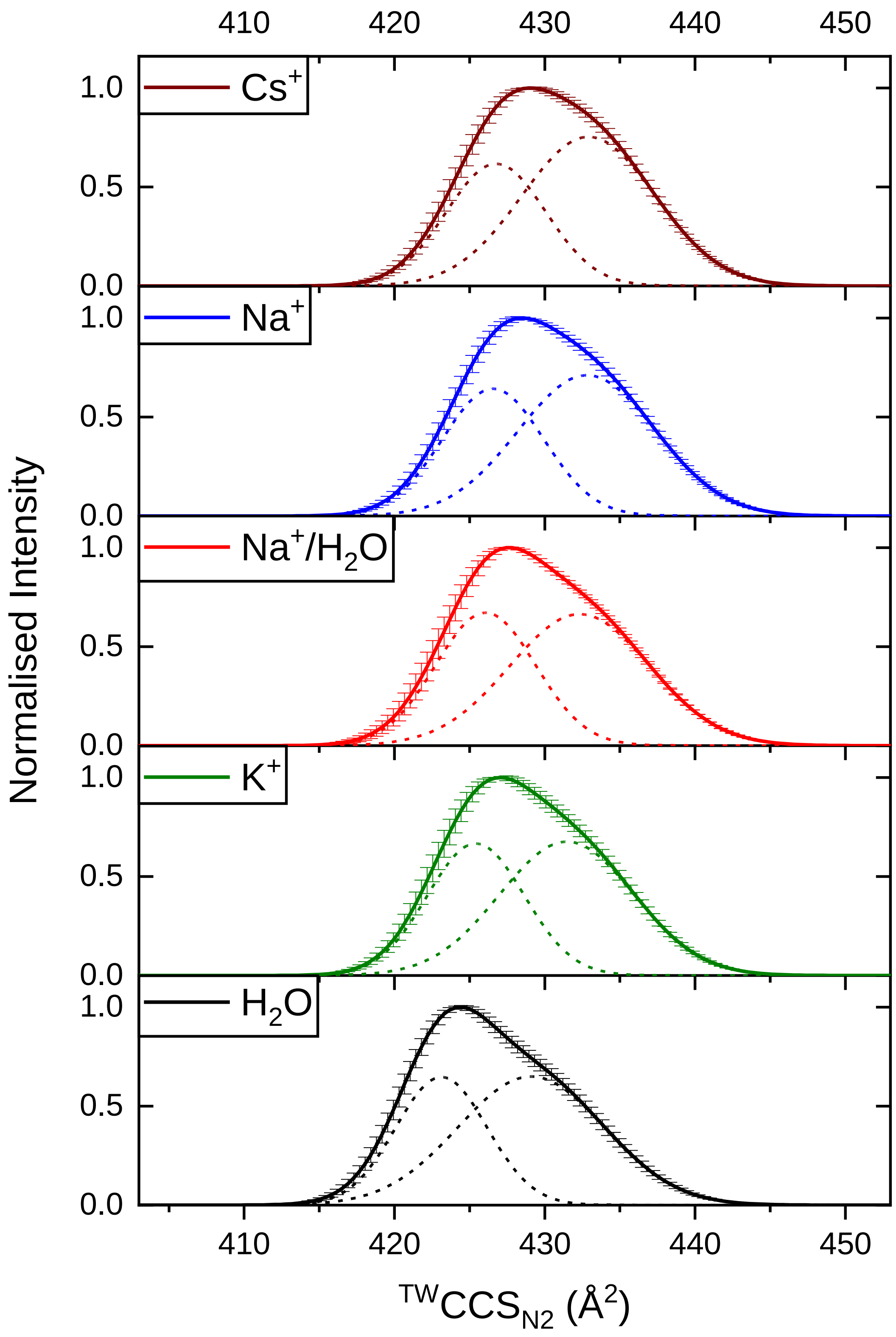

Figure S3: $^{TW}CCS_{N2}$ distributions of [**Ring$_{Cr}$** + A]$^+$ (A^+ = Na^+, Na^+ + H_2O, K^+, Cs^+) and [**Ring$_{Cr}$** + H_2O]$^+$ including experimental error. All distributions are fitted with two Gaussian distributions and averaged over four datasets, except for [**Ring$_{Cr}$** + H_2O]$^+$, for which two sets were averaged.

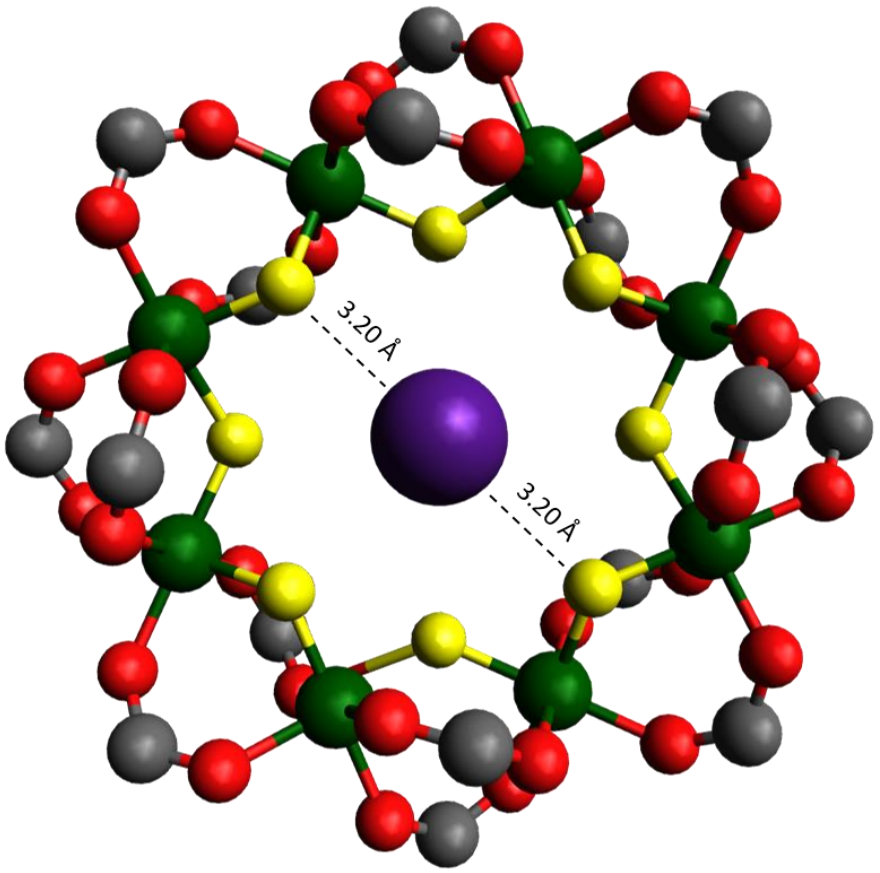

Figure S4: DFT-optimized structure of [**Ring$_{Cr}$** + Cs]$^+$ (Cs: purple, Cr: green, F: yellow, O: red, C: grey; *tert*-butyl groups were omitted for clarity). The shortest Cs-F distance was enumerated along with the Cs-F distance on the opposite side. Optimized coordinates, ESP charges and minimum energy can be found in the Supplementary Dataset.

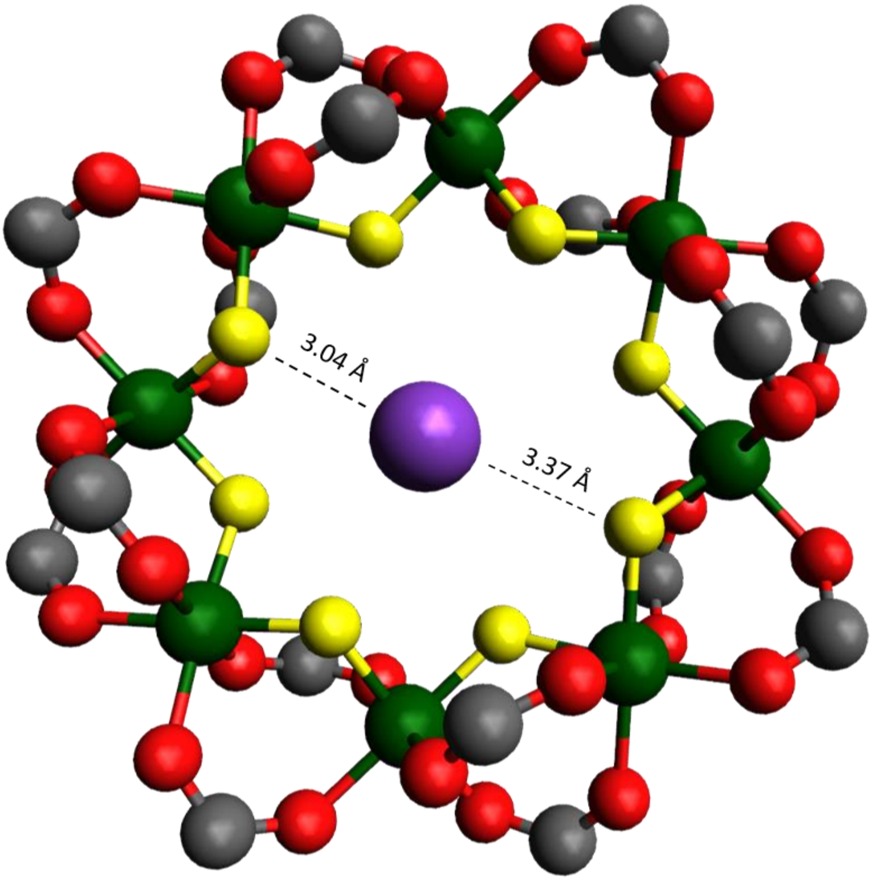

Figure S5: DFT-optimized structure of [**Ring**$_{\mathbf{Cr}}$ + K]$^+$ (K: purple, Cr: green, F: yellow, O: red, C: grey; *tert*-butyl groups were omitted for clarity). The shortest K-F distance was enumerated along with the K-F distance on the opposite side. Optimized coordinates, ESP charges and minimum energy can be found in the Supplementary Dataset.

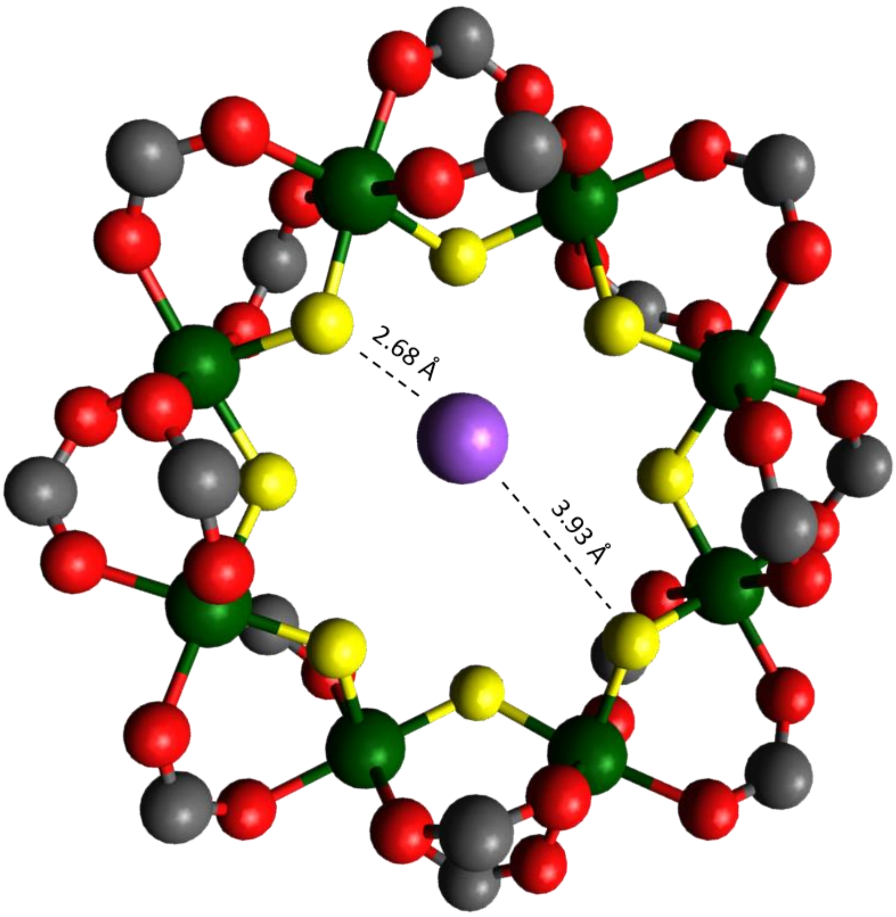

Figure S6: DFT-optimized structure of [**Ring$_{Cr}$** + Na]$^+$ (Na: purple, Cr: green, F: yellow, O: red, C: grey; *tert*-butyl groups were omitted for clarity). The shortest Na-F distance was enumerated along with the Na-F distance on the opposite side. Optimized coordinates, ESP charges and minimum energy can be found in the Supplementary Dataset.

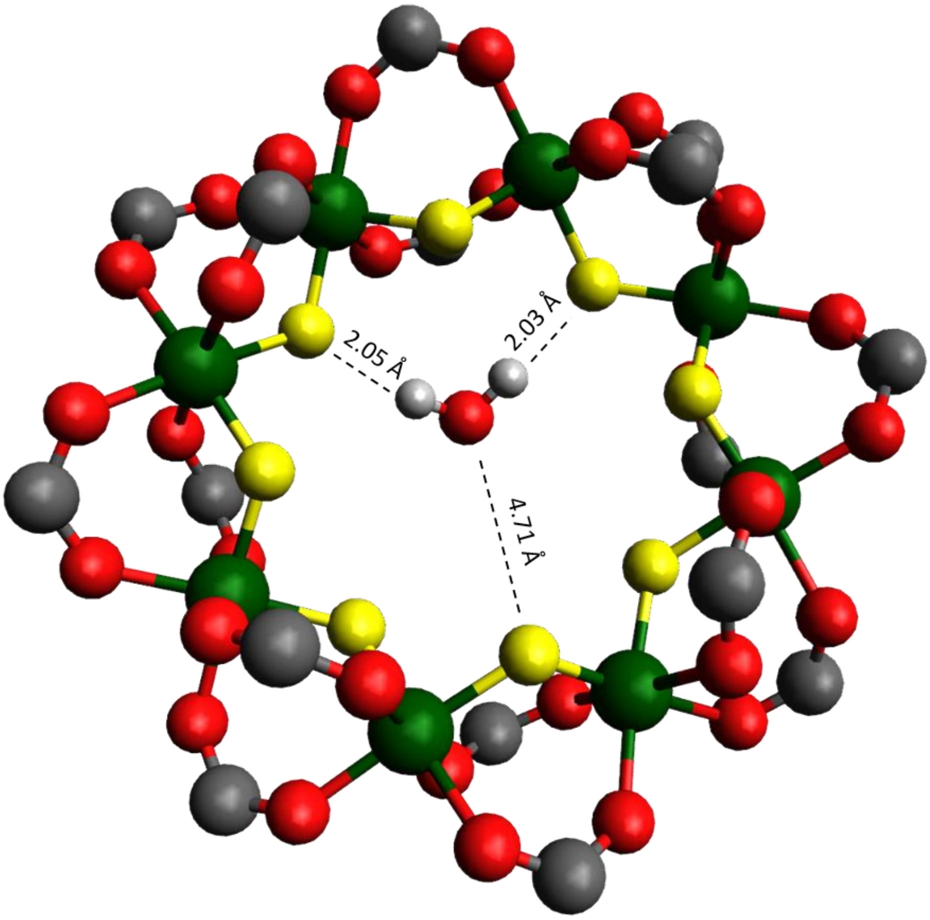

Figure S7: DFT-optimized structure of [**Ring**$_{\mathbf{Cr}}$ + H_2O]$^+$ (Cr: green, F: yellow, O: red, C: grey, H: white; *tert*-butyl groups were omitted for clarity). The H-F hydrogen bond lengths were enumerated along with the distance between the water oxygen and the furthest fluoride. Optimized coordinates, ESP charges, the minimum energy and the spin-density analysis can be found in the Supplementary Dataset.

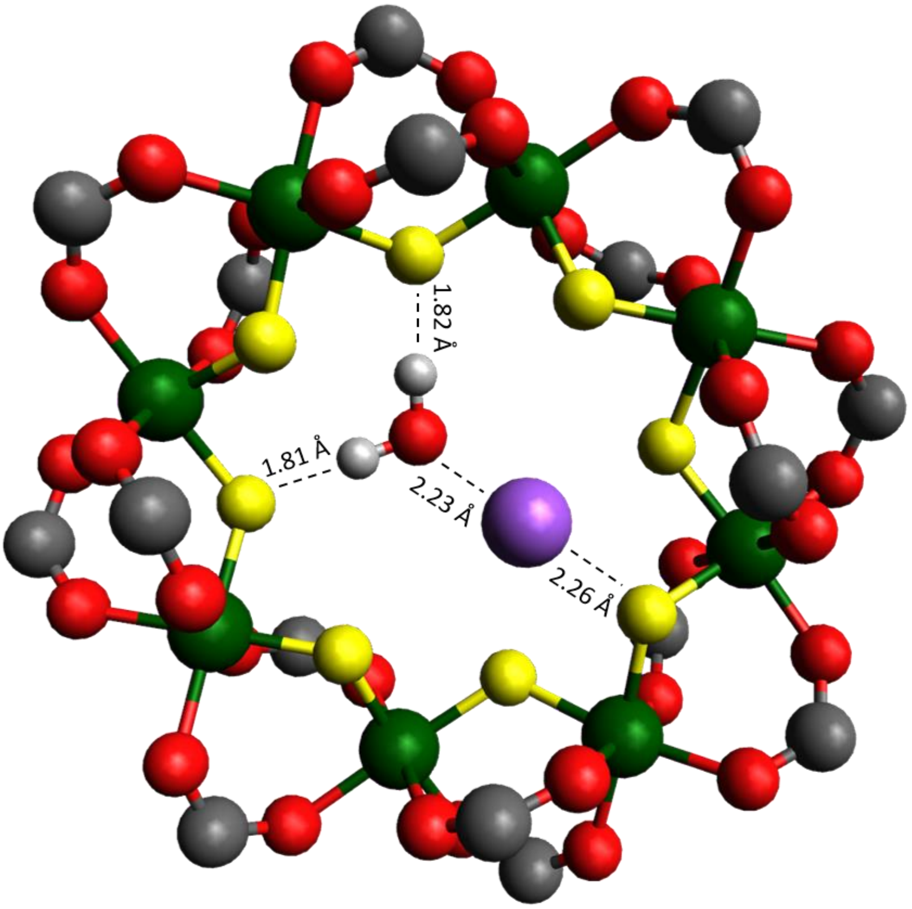

Figure S8: DFT-optimized structure of [**Ring$_{Cr}$** + Na + H_2O]$^+$ (Na: purple, Cr: green, F: yellow, O: red, C: grey, H: white; *tert*-butyl groups were omitted for clarity). The H-F hydrogen bond lengths were enumerated along with the distances between the water oxygen and Na^+ as well as Na^+ and the closest F^-. Optimized coordinates, ESP charges and the minimum energy can be found in the Supplementary Dataset.

Table S1: $^{TM}CCS_{N2}$ and $^{TW}CCS_{N2}$ values of [**Ring**$_{\mathbf{Cr}}$ + A]$^+$ (A^+ = Na^+, Na^+ + H_2O, K^+, Cs^+) and [**Ring**$_{\mathbf{Cr}}$ + H_2O]$^+$ including error (TW = "Traveling Wave", TM = "Trajectory Method" computed with IMoS[12]).

**Cationic Adduct**	$^{TW}CCS_{N2}$ **(Å²)**	$^{TM}CCS_{N2}$ **(Å²)**
[**Ring**$_{\mathbf{Cr}}$ + H_2O]$^+$	426.3 ± 1.0	467.9 ± 1.2
[**Ring**$_{\mathbf{Cr}}$ + Na]$^+$	430.0 ± 0.8	467.0 ± 1.5
[**Ring**$_{\mathbf{Cr}}$ + Na + H_2O]$^+$	429.4 ± 0.4	468.2 ± 1.7
[**Ring**$_{\mathbf{Cr}}$ + K]$^+$	428.6 ± 1.1	466.8 ± 1.5
[**Ring**$_{\mathbf{Cr}}$ + Cs]$^+$	430.3 ± 0.9	464.2 ± 1.2

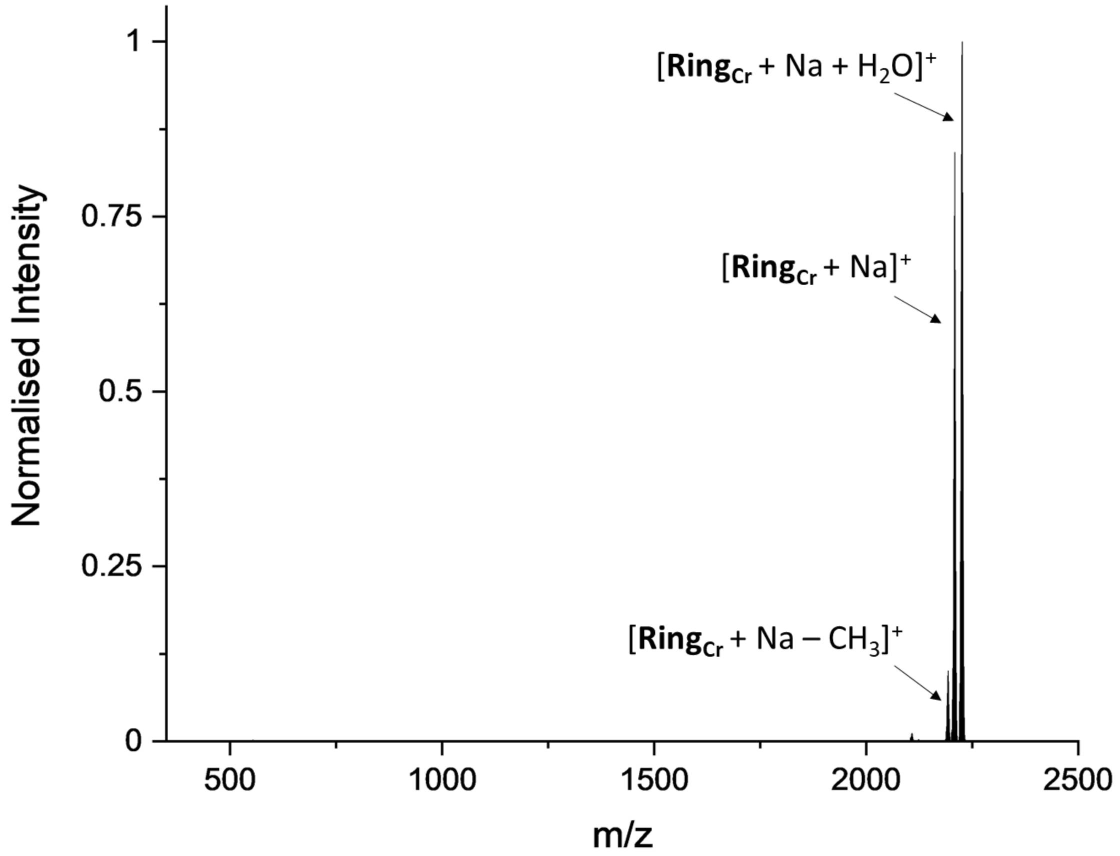

Figure S9: MS^2 spectrum of [**Ring**$_{Cr}$ + Na + H_2O]$^+$ at E_{lab} = 0 eV (no collisional activation). 2 µM **Ring**$_{Cr}$ were used in 4:1 toluene/methanol and 500 µM NaI. [**Ring**$_{Cr}$ + Na + H_2O]$^+$ was selected at *m/z* = 2226. Observed ions are labelled.

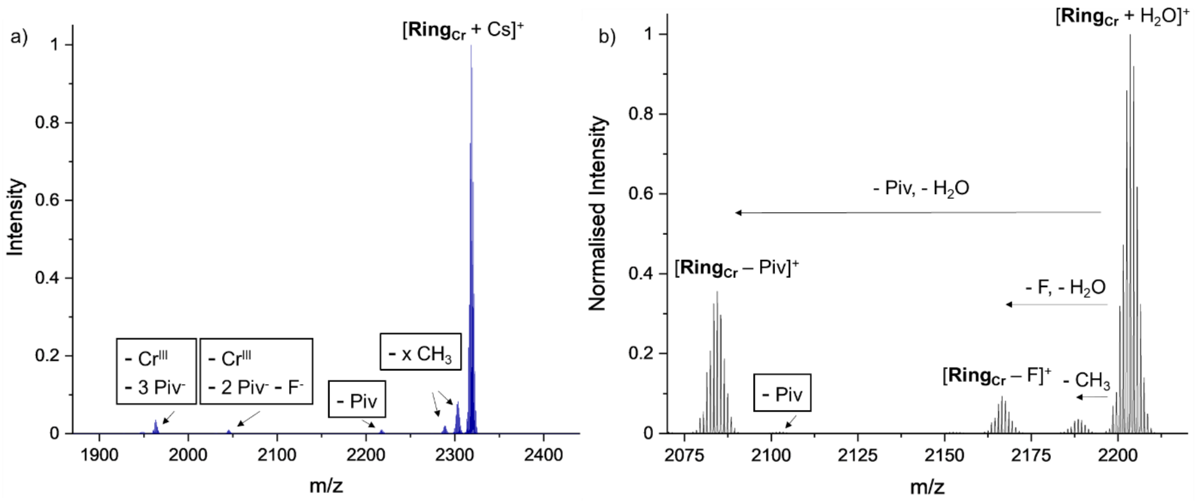

Figure S10: MS^2 spectra of a) [**Ring$_{Cr}$** + Cs]$^+$ at E_{lab} = 190 eV and b) [**Ring$_{Cr}$** + H_2O]$^+$ at E_{lab} = 110 eV. 2 µM **Ring$_{Cr}$** were used in 4:1 toluene/methanol and a) 500 µM CsI or b) 1% HCOOH. Ions were isolated at a) *m/z* = 2318 and b) *m/z* = 2203. Losses of methyl groups as well as the loss of a single, likely neutral pivalate ligand were observed as minor fragmentation channels for all studied adducts [**Ring$_{Cr}$** + A]$^+$ (A^+ = Na^+, Na^+ + H_2O, K^+, Cs^+) and [**Ring$_{Cr}$** + H_2O]$^+$.

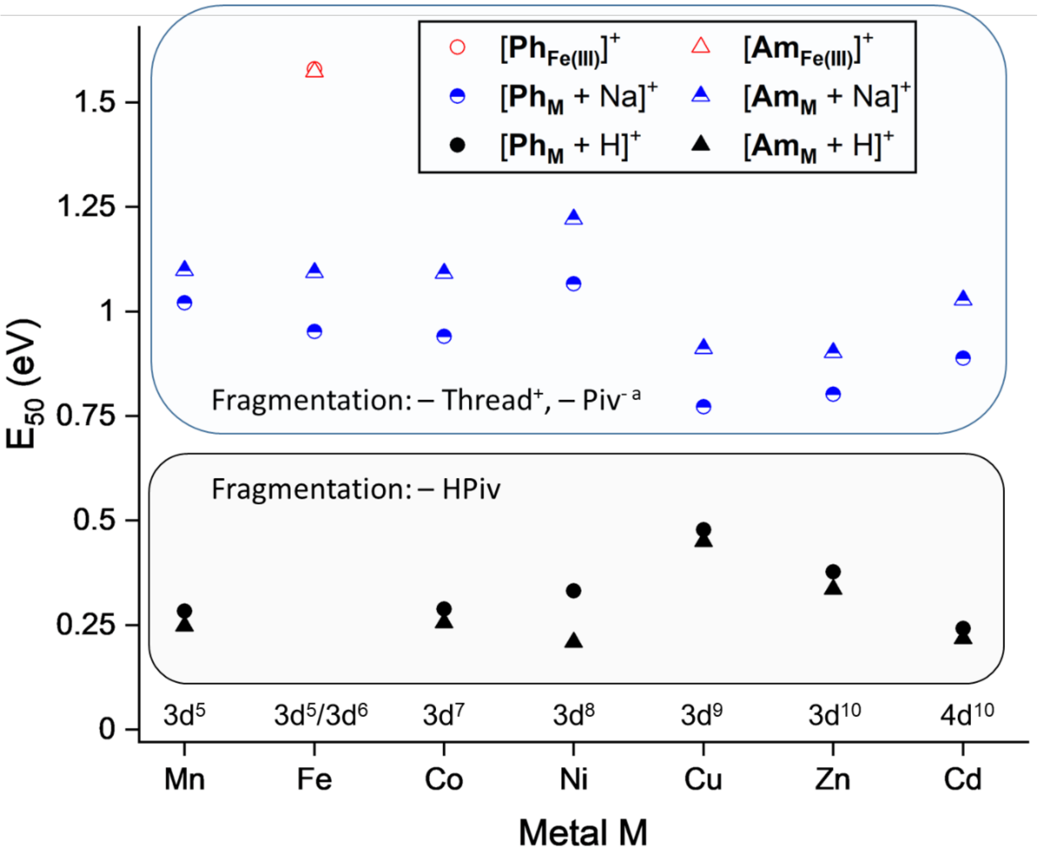

Figure S11: E_{50} values of [**Ph**$_{M}$ + A]$^{+}$ and [**Am**$_{M}$ + A]$^{+}$ (A^{+} = H^{+}, Na^{+}) with respect to M, as well as the E_{50} values of the oxidised Fe^{III} species [**Ph**$_{Fe(III)}$]$^{+}$ and [**Am**$_{Fe(III)}$]$^{+}$. [a]For [**Am**$_{Cu}$ + Na]$^{+}$, the main dissociation channel involves the loss of Cu^{II} and 2 Piv^{-}. Error bars in all cases are smaller than the symbol size, but are omitted for clarity (Table 2). The electron configuration of Fe depends on the oxidation state ($3d^5$: Fe^{III}, $3d^6$: Fe^{II}). Reproduced with permission from American Chemical Society, Copyright © 2022 from Ref. 3.

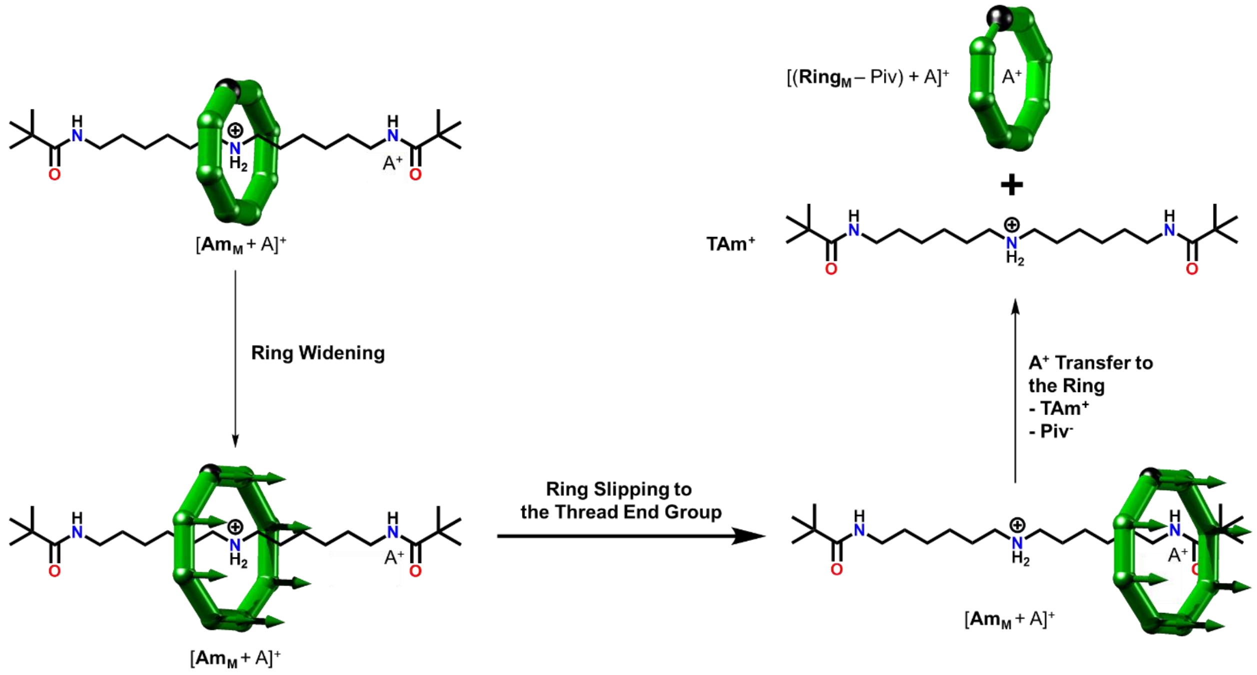

Figure S12: Suggested disassembly mechanism of [**Am$_M$** + A]$^+$ (A$^+$ = Na$^+$, K$^+$, Cs$^+$). The widened ring moves to the amide end groups of **TAm$^+$**, where A$^+$ is transferred from the thread end group to the ring. Deslipping of the thread and loss of an anionic pivalate ligand leads to the product ions [(**Ring$_M$** – Piv) + A]$^+$ and **TAm$^+$**.

Table S2: $^{TW}CCS_{N2}$ values of [**Am**$_M$ + A]$^+$ and [**Ph**$_M$ + A]$^+$ (A^+ = H^+, Na^+) including experimental error.

$^{TW}CCS_{N2}$ (Å^2)	**Mn^{II}**	**Fe^{II}**	**Co^{II}**	**Ni^{II}**	**Cu^{II}**	**Zn^{II}**	**Cd^{II}**
[**Am**$_M$ + H]$^+$	480.2 ± 0.7	-	487.7 ± 0.3	484.0 ± 1.1	493.1 ± 0.1	490.9 ± 0.5	480.3 ± 0.1
[**Am**$_M$ + Na]$^+$	483.6 ± 1.3	483.1 ± 1.4	480.9 ± 1.1	482.0 ± 0.6	482.6 ± 0.1	481.7 ± 0.7	486.6 ± 0.8
[**Am**$_M$ + K]$^+$	-	-	486.3 ± 1.1	-	-	-	-
[**Am**$_M$ + Cs]$^+$	-	-	490.5 ± 0.1	-	-	-	-
[**Ph**$_M$ + H]$^+$	445.9 ± 0.5	-	444.3 ± 1.2	444.5 ± 0.8	-	-	447.6 ± 0.5
[**Ph**$_M$ + Na]$^+$	446.2 ± 0.7	445.3 ± 1.3	444.3 ± 1.1	444.2 ± 0.6	445.1 ± 0.7	443.6 ± 0.6	448.1 ± 0.2
[**Ph**$_M$ + K]$^+$	447.7 ± 0.5	-	-	-	-	-	-
[**Ph**$_M$ + Cs]$^+$	450.3 ± 1.4	-	-	-	-	-	-

Table S3: *FWHM* ($^{TW}CCS_{N2}$) values of [$\mathbf{Am_M}$ + A]$^+$ and [$\mathbf{Ph_M}$ + A]$^+$ ($A^+ = H^+$, Na^+) including experimental error.

***FWHM* ($^{TW}CCS_{N2}$) (Å^2)**	**Mn^{II}**	**Fe^{II}**	**Co^{II}**	**Ni^{II}**	**Cu^{II}**	**Zn^{II}**	**Cd^{II}**
[$\mathbf{Am_M}$ + H]$^+$	10.3 ± 0.8	-	10.8 ± 0.8	10.5 ± 0.2	10.4 ± 1.0	10.7 ± 1.4	9.6 ± 0.8
[$\mathbf{Am_M}$ + Na]$^+$	10.4 ± 1.2	9.0 ± 0.4	10.7 ± 0.7	10.8 ± 0.1	10.5 ± 0.7	11.1 ± 1.3	9.0 ± 0.2
[$\mathbf{Am_M}$ + K]$^+$	-	-	12.2 ± 0.3	-	-	-	-
[$\mathbf{Am_M}$ + Cs]$^+$	-	-	13.4 ± 0.3	-	-	-	-
[$\mathbf{Ph_M}$ + H]$^+$	9.6 ± 0.4	-	10.8 ± 0.9	10.7 ± 0.6	-	-	10.2 ± 0.6
[$\mathbf{Ph_M}$ + Na]$^+$	10.4 ± 0.6	11.7 ± 2.1	10.9 ± 1.4	10.7 ± 0.9	11.4 ± 0.9	11.1 ± 0.1	10.1 ± 0.3
[$\mathbf{Ph_M}$ + K]$^+$	10.5 ± 0.6	-	-	-	-	-	-
[$\mathbf{Ph_M}$ + Cs]$^+$	11.1 ± 0.5	-	-	-	-	-	-

References

(1) Vitórica-Yrezábal, I. J.; Sava, D. F.; Timco, G. A.; Brown, M. S.; Savage, M.; Godfrey, H. G. W.; Moreau, F.; Schröder, M.; Siperstein, F.; Brammer, L.; Yang, S.; Attfield, M. P.; McDouall, J. J. W.; Winpenny, R. E. P. Binding CO_2 by a Cr_8 Metallacrown. *Angew. Chem., Int. Ed.* **2017**, *56* (20), 5527–5530. https://doi.org/10.1002/anie.201701726.

(2) Ballesteros, B.; Faust, T. B.; Lee, C. F.; Leigh, D. A.; Muryn, C. A.; Pritchard, R. G.; Schultz, D.; Teat, S. J.; Timco, G. A.; Winpenny, R. E. P. Synthesis, Structure, and Dynamic Properties of Hybrid Organic-Inorganic Rotaxanes. *J. Am. Chem. Soc.* **2010**, *132* (43), 15435–15444. https://doi.org/10.1021/ja1074773.

(3) Geue, N.; Bennett, T. S.; Arama, A. A.; Ramakers, L. A. I.; Whitehead, G. F. S.; Timco, G. A.; Armentrout, P. B.; Mcinnes, E. J. L.; Burton, N. A.; Winpenny, R. E. P.; Barran, P. E. Disassembly Mechanisms and Energetics of Polymetallic Rings and Rotaxanes. *J. Am. Chem. Soc.* **2022**, *144* (49), 22528–22539. https://doi.org/10.1021/jacs.2c07522.

(4) Fort, K. L.; Van De Waterbeemd, M.; Boll, D.; Reinhardt-Szyba, M.; Belov, M. E.; Sasaki, E.; Zschoche, R.; Hilvert, D.; Makarov, A. A.; Heck, A. J. R. Expanding the Structural Analysis Capabilities on an Orbitrap-Based Mass Spectrometer for Large Macromolecular Complexes. *Analyst* **2018**, *143* (1), 100–105. https://doi.org/10.1039/c7an01629h.

(5) Giles, K.; Ujma, J.; Wildgoose, J.; Pringle, S.; Richardson, K.; Langridge, D.; Green, M. A Cyclic Ion Mobility-Mass Spectrometry System. *Anal. Chem.* **2019**, *91* (13), 8564–8573. https://doi.org/10.1021/acs.analchem.9b01838.

(6) Ruotolo, B. T.; Benesch, J. L. P.; Sandercock, A. M.; Hyung, S. J.; Robinson, C. V. Ion Mobility-Mass Spectrometry Analysis of Large Protein Complexes. *Nat. Protoc.* **2008**, *3* (7), 1139–1152. https://doi.org/10.1038/nprot.2008.78.

(7) Stow, S. M.; Causon, T. J.; Zheng, X.; Kurulugama, R. T.; Mairinger, T.; May, J. C.; Rennie, E. E.; Baker, E. S.; Smith, R. D.; McLean, J. A.; Hann, S.; Fjeldsted, J. C. An Interlaboratory Evaluation of Drift Tube Ion Mobility-Mass Spectrometry Collision Cross Section Measurements. *Anal. Chem.* **2017**, *89* (17), 9048–9055. https://doi.org/10.1021/acs.analchem.7b01729.

(8) Frisch, M. J.; Trucks, G. W.; Schlegel, H. B.; Scuseria, G. E. .; Robb, M. A. .; Cheeseman, J. R. .; Scalmani, G. .; Barone, V. .; Petersson, G. A. .; Nakatsuji, H. .; Li, X. .; Caricato, M. .; Marenich, A. V. .; Bloino, J. .; Janesko, B. G. .; Gomperts, R. .; Mennucci, B. .; Hratchian, H. P. .; Ortiz, J. V. .; Izmaylov, A. F. .; Sonnenberg, J. L. .; Williams-Young, D. .; Ding, F. .; Lipparini, F. .; Egidi, F. .; Goings, J. .; Peng, B. .; Petrone, A. .; Henderson, T. .; Ranasinghe, D. .; Zakrzewski, V. G. .; Gao, J. .; Rega, N. .; Zheng, G. .; Liang, W. .; Hada, M. .; Ehara, M. .; Toyota, K. .; Fukuda, R. .; Hasegawa, J. .; Ishida, M. .; Nakajima, T. .; Honda, Y. .; Kitao, O. .; Nakai, H. .; Vreven, T. .; Throssell, K. .; Montgomery, J. A. J. .; Peralta, J. E. .; Ogliaro, F. .; Bearpark, M. J. .; Heyd, J. J. .; Brothers, E. N. .; Kudin, K. N. .; Staroverov, V. N. .; Keith, T. A. .; Kobayashi, R. .; Normand, J. .; Raghavachari, K. .; Rendell, A. P. .; Burant, J. C. .; Iyengar, S. S. .; Tomasi, J. .; Cossi, M. .; Millam, J. M. .; Klene, M. .; Adamo, C. .; Cammi, R. .; Ochterski, J. W. .; Martin, R. L. .; Morokuma, K. .; Farkas, O. .; Foresman, J. B. .; Fox, D. J. Gaussian 16, Revision C.01. Gaussian, Inc.: Wallingford CT 2016.

(9) Grimme, S.; Antony, J.; Ehrlich, S.; Krieg, H. A Consistent and Accurate Ab Initio Parametrization of Density Functional Dispersion Correction (DFT-D) for the 94 Elements H-Pu. *J. Chem. Phys.* **2010**, *132* (15), 154104. https://doi.org/10.1063/1.3382344.

(10) Hay, P. J.; Wadt, W. R. Ab Initio Effective Core Potentials for Molecular Calculations. Potentials for K to Au Including the Outermost Core Orbitale. *J. Chem. Phys.* **1985**, *82* (1), 299–310. https://doi.org/10.1063/1.448975.

(11) Glendening, E. D.; Reed, A. E.; Carpenter, J. E.; Weinhold, F. NBO Version 3.1.

(12) Shrivastav, V.; Nahin, M.; Hogan, C. J.; Larriba-Andaluz, C. Benchmark Comparison for a Multi-Processing Ion Mobility Calculator in the Free Molecular Regime. *J. Am. Soc. Mass Spectrom.* **2017**, *28* (8), 1540–1551. https://doi.org/10.1007/s13361-017-1661-8.
